# The infected diabetic foot: Risk factors for re‐infection after treatment for diabetic foot osteomyelitis

**DOI:** 10.1111/wrr.13246

**Published:** 2025-01-21

**Authors:** Lawrence A. Lavery, Mario C. Reyes, Bijan Najafi, Tyler L. Coye, Matthew Sideman, Michael C. Siah, Arthur N. Tarricone

**Affiliations:** ^1^ Department of Plastic Surgery University of Texas Southwestern Medical Center Dallas Texas USA; ^2^ Department of Orthopedic Surgery University of Texas Health Science Center San Antonio Texas USA; ^3^ Department of Surgery Baylor College of Medicine Houston Texas USA; ^4^ Department of Surgery University of Texas Health Science Center San Antonio Texas USA; ^5^ Department of Surgery University of Texas Southwestern Medical Center Dallas Texas USA

**Keywords:** amputation, diabetes, infection, osteomyelitis, ulcer

## Abstract

Our objective was to evaluate risk factors for re‐infection in patients after treatment for diabetic foot osteomyelitis (OM). We used pooled patient level data from two RTCs that evaluated patients with diabetic foot infections. We evaluated 171 patients with OM. OM was confirmed with bone culture or histopathology. Data from the 12‐month follow‐up were used to determine clinical outcomes. Re‐infection occurred in 47 (27.5%) patients. Risk factors for re‐infection were Toe Brachial Index <0.40 (25.7% vs. 9.8%, *p* = 0.02), skin perfusion pressure <40 mmHg (6.3% vs. 5.9%, *p* = 0.04), wound healing (55.3% vs. 75.0%, *p* = 0.01), time to heal (156.0, 69.5–365 vs. 91.5, 38.8–365, *p* = 0.001), and history of MI (14.9% vs. 3.2%, *p* = 0.005). During 12‐month follow‐up, patients with re‐infections were 198.8 times more likely to require a foot related hospitalisation (81.8% vs. 0.0%, *p* = 0.001), 10.4 times more likely have an all‐cause hospitalisation (70.2% vs. 18.5%, *p* = 0.001) and 9.4 times more likely to need an amputation (36.2% vs. 5.6%, *p* = 0.001). Patients with re‐infection had a significantly longer median length of hospitalisation (20.0, 13.5–34.5 vs. 14.0, 10.0–22.0, *p* = 0.003) and median length of antibiotic duration (55.0, 35.0–87.0 vs. 46.0, 22.8–68.0, *p* = 0.03). Patients with re‐infection are less likely to heal and have more foot‐related hospitalizations and amputations.

**Abbreviations**
OM: osteomyelitis
SPP: skin perfusion pressure
TBI: toe brachial index

## 1 INTRODUCTION

Patients with diabetic foot osteomyelitis have higher rates of amputation, higher levels of amputation and more surgeries, longer hospitalizations and longer duration of antibiotic treatment compared to patients with soft tissue infections.^1^, ^2^, ^3^ Many studies do not report long term outcomes such as wound healing, re‐ulceration, re‐infection, and hospital readmission. The incidence of re‐infection ranges from 16.7% to 56.7% per year.^4^, ^5^, ^6^ Re‐infection during the year following the initial hospitalisation is about twice as high amongst people with osteomyelitis.^7^ For most patients, re‐infection is the underlying cause that leads to additional hospitalizations, parenteral antibiotic exposure, and amputations. We could not identify any studies that evaluate risk factors for re‐infection in diabetic patients treated for osteomyelitis or soft tissue infection. The goal of this study was to evaluate risk factors for re‐infection in patients previously treated for diabetic foot osteomyelitis and clinical outcomes associated with re‐infection.

## 2 METHODS

This study was approved by the Institutional Review Board at our institutions. We performed a post hoc analysis using data from two randomised controlled trials by our group that used the same evaluation criteria and operational definitions for outcomes and adverse events.^5^, ^6^ In the RCTs, we enrolled 240 patients with moderate and severe diabetic foot infections using the criteria defined by IWGDF between the age of 18 and 89. For this post hoc analysis, we included patients with osteomyelitis who had 12‐month follow‐up or died. All the study subjects underwent surgery for infection. The initial diagnosis of osteomyelitis was confirmed by bone biopsy with positive bone culture or bone histopathology.^8^ Absence of osteomyelitis was determined by a negative MRI, negative SPECT CT, or negative bone biopsy (both histology and culture).^9^, ^10^, ^11^ After the initial incision and drainage or amputation, the surgical site was irrigated with normal saline, and bone cultures were obtained using clean instruments. We categorised patients in two groups, with no residual osteomyelitis (NRO) and those with incomplete excision of infection bone with residual osteomyelitis (RO). Infected wounds were left open and taken back to the operating theatre in 48–72 h. Successful treatment of osteomyelitis was defined as no bone re‐infection at the same site in the year following the index hospitalisation.

Sensory neuropathy was noted when there was abnormal vibration sensation (VPT Salix Medical, San Antonio, TX) or any missed sites with the 10‐gram Semmes‐Weinstein monofilament. Peripheral arterial disease was defined as an ankle to arm systolic blood pressure ratio (ABI) of <0.90.^12^ Non‐compressible artery was defined as an ABI greater than 1.30. Furthermore, we measured skin perfusion pressure measurements (SPP) on the plantar and dorsal aspects of the involved foot (Sensilase, Väsamed, Eden Prairie, MN Device). Foot ulceration was defined as full‐thickness skin lesions involving any portion of the foot or ankle, and foot infection severity was defined according to the criteria of the IWGDF.^13^ Wound healing was defined as complete epithelialization with no drainage.

Categorical variables were described as frequency and percentage whilst continuous variables were reported as mean and standard deviation. Differences in patient characteristics between patients with re‐infection and no re‐infection were calculated with chi‐squared test of homogeneity or Fisher exact test for categorical variables, and Mann–Whitney *U* test for continuous variables. Risk factors for adverse outcomes were calculated using relative risk analysis. Survival time to event analysis using Kaplan Meir plot was censored at 365 days for healing.

## 3 RESULTS

A total of 240 patients with moderate to severe foot infections were initially evaluated. This study included 171 patients with diabetic foot osteomyelitis (71.3%) with a minimum follow‐up of at least 12 months. The incidence of re‐infection was 27.5% (*n* = 47) per year. There were few differences in the baseline characteristics of people that experienced re‐infection compared to those with no re‐infection (Table 1).

**TABLE 1:** Risk factors for re‐infection in patients with diabetic foot osteomyelitis.

	Reinfection (*n* = 47)	No reinfection (*n* = 124)	(95% CI)	*p*‐value
Age	52.0 (10.1)	51.5 (9.7)	−2.8 to 3.8	0.76
Male	37 (78.7)	101 (81.5)	0.8 (0.3–1.9)	0.69
BMI (kg/m^2^)	31.6 (7.5)	31.3 (7.7)	−2.2 to 2.9	0.77
Race				
Non‐Hispanic White	13 (27.7)	24 (19.4)	1.6 (0.7–3.4)	0.24
African Descent	11 (23.4)	43 (34.7)	0.5 (0.3–1.2)	0.16
Hispanic	22 (46.8)	56 (45.2)	1.1 (0.5–2.1)	0.85
Social factors				
<12 years of education	26 (55.3)	66 (53.2)	1.1 (0.5–2.1)	0.80
Spanish language	5 (10.6)	29 (23.4)	0.4 (0.1–1.1)	0.06
Living Alon	9 (19.1)	13 (10.5)	2.0 (0.8–5.1)	0.14
Household ambulatory	0 (0.0)	6 (4.8)	0.2 (0.0–3.5)	0.26
Married	17 (36.2)	38 (30.6)	1.2 (0.6–2.6)	0.49
Tobacco use	10 (21.3)	26 (21.0)	1.0 (0.4–2.3)	0.96
Alcohol use	8 (17.0)	33 (26.6)	0.6 (0.2–1.2)	0.19
Illicit drug use	1 (2.1)	7 (5.6)	0.4 (0.1–3.0)	0.33
Medical history				
Hypertension	35 (74.5)	95 (76.6)	0.8 (0.4–1.9)	0.77
MI history	7 (14.9)	4 (3.2)	5.3 (1.4–18.9)	0.005
CHF	7 (14.9)	12 (9.7)	1.6 (0.6–4.4)	0.33
HIV	0 (0.0)	2 (1.6)	0.5 (0.0–10.9)	0.38
Retinopathy	7 (14.9)	23 (18.5)	0.8 (0.3–1.9)	0.58
CKD I–IV	14 (29.9)	26 (21.0)	1.6 (0.7–3.4)	0.22
ESRD	2 (4.3)	9 (7.3)	0.6 (0.1–2.7)	0.48
Sensory neuropathy	46 (97.9)	120 (97.6)	1.1 (0.1–11.3)	0.9
Abnormal monofilament	43 (93.5)	106 (87.6)	2.0 (0.6–7.4)	0.27
VPT forefoot	52.3 (23.2)	48.2 (23.8)	−3.9 to 12.1	0.32
Charcot arthropathy history	2 (4.3)	2 (4.8)	0.9 (0.2–4.4)	0.87
Prior amputation	28 (59.6)	55 (44.4)	1.8 (0.9–3.6)	0.08
Medications				
Insulin	33 (70.2)	78 (62.9)	1.4 (0.6)	0.37
Calcium channel blockers	13 (27.7)	31 (25.0)	1.1 (0.5–2.4)	0.72
Steroids	5 (10.6)	8 (6.5)	1.7 (0.5–5.5)	0.36
Beta‐blockers	11 (23.4)	26 (21.0)	1.1 (0.5–2.5)	0.73
Gabapentin	15 (31.9)	37 (29.8)	1.1 (0.5–2.3)	0.79
Pre‐gabalin	0 (0.0)	7 (5.6)	0.1 (0.0–2.9)	0.22
Admission characteristics				
SIRS criteria	11 (23.4)	25 (20.2)	1.2 (0.5–2.7)	0.64
Temperature > 38.6	7 (14.9)	14 (11.3)	1.3 (0.5–3.7)	0.52
Heart rate > 90	17 (36.2)	51 (41.1)	0.8 (0.4–1.6)	0.55
Respiratory rate > 20	8 (17.0)	10 (8.1)	2.3 (0.9–6.3)	0.09
WBC > 12,000	16 (34.8)	36 (29.0)	1.3 (0.6–2.7)	0.47
Admissions labs				
CRP	11.7 (14.3)	11.5 (10.2)	−3.8 to 4.0	0.95
ESR	78.8 (38.2)	78.2 (40.6)	−13.0 to 14.3	0.93
Glycated haemoglobin (%)	9.6 (2.6)	9.5 (2.8)	−0.9 to 0.9	0.87
eGFR	50.2 (16.0)	52.9 (15.1)	−7.9 to 2.5	0.31
Wound characteristics				
Ulcer duration Median (IQR)	34.0 (15.5–90.0)	30.0 (10.0–60.0)		0.37
Wound area (cm^2^) Median (IQR)	10.8 (4.6–19.2)	11.4 (6.6–19.8)		0.43
Wound volume (cm^3^) Median (IQR)	7.2 (2.3–17.0)	8.9 (2.5–20.2)		0.66
Ankle Brachial Index				
<0.90	8 (17.0)	9 (7.3)	2.6 (0.9–7.2)	0.06
0.90–1.30	27 (57.4)	75 (61.0)	0.8 (0.4–1.7)	0.67
>1.30	12 (25.5)	39 (31.7)	0.7 (0.3–1.5)	0.43
Toe Brachial Index (*n* = 137)	0.7 (0.3)	0.7 (0.2)	−0.2‐0.4	0.25
TBI < 0.6	13 (37.1)	35 (34.3)	1.1 (0.5–2.5)	0.76
TBI < 0.4	9 (25.7)	10 (9.8)	3.1 (1.2–8.6)	0.02
SPP Dorsal Medial	62.3 (28.6)	59.8 (21.9)	−7.3‐12.3	0.61
SPP Dorsal Medial ≤ 40 mmHg	12 (27.9)	24 (20.5)	1.6 (0.7–3.6)	0.24
SPP Dorsal Lateral	67.3 (21.1)	62.3 (24.3)	−3.3‐13.4	0.24
SPP Dorsal Lateral ≤ 40 mmHg	4 (9.3)	24 (20.5)	0.4 (0.1–1.3)	0.12
SPP Plantar Medial	72.4 (23.4)	74.7 (22.7)	−10.4‐5.7	0.57
SPP Plantar Medial ≤ 40 mmHg	4 (9.3)	8 (6.8)	1.4 (0.4–4.9)	0.60
SPP Plantar Lateral	72.3 (26.7)	79.3 (23.5)	−15.6‐1.6	0.11
SPP Plantar Lateral ≤ 40 mmHg	7 (16.3)	7 (5.9)	3.0 (1.0–9.1)	0.04
Monckeberg's sclerosis	26 (55.3)	77 (58.9)	0.9 (0.4–1.7)	0.68
Residual osteomyelitis	27 (57.4)	65 (52.4)	0.8 (0.4–1.6)	0.56
Wound healed	26 (55.3)	93 (75.0)	0.4 (0.2–0.8)	0.01
Wound healing time	156.0 (69.5–365)	91.5 (38.8–365)		0.03

*Note*: Dichotomous variables presented as *N* (%). Continuous variables presented as mean (standard deviation).

**FIGURE 1:**
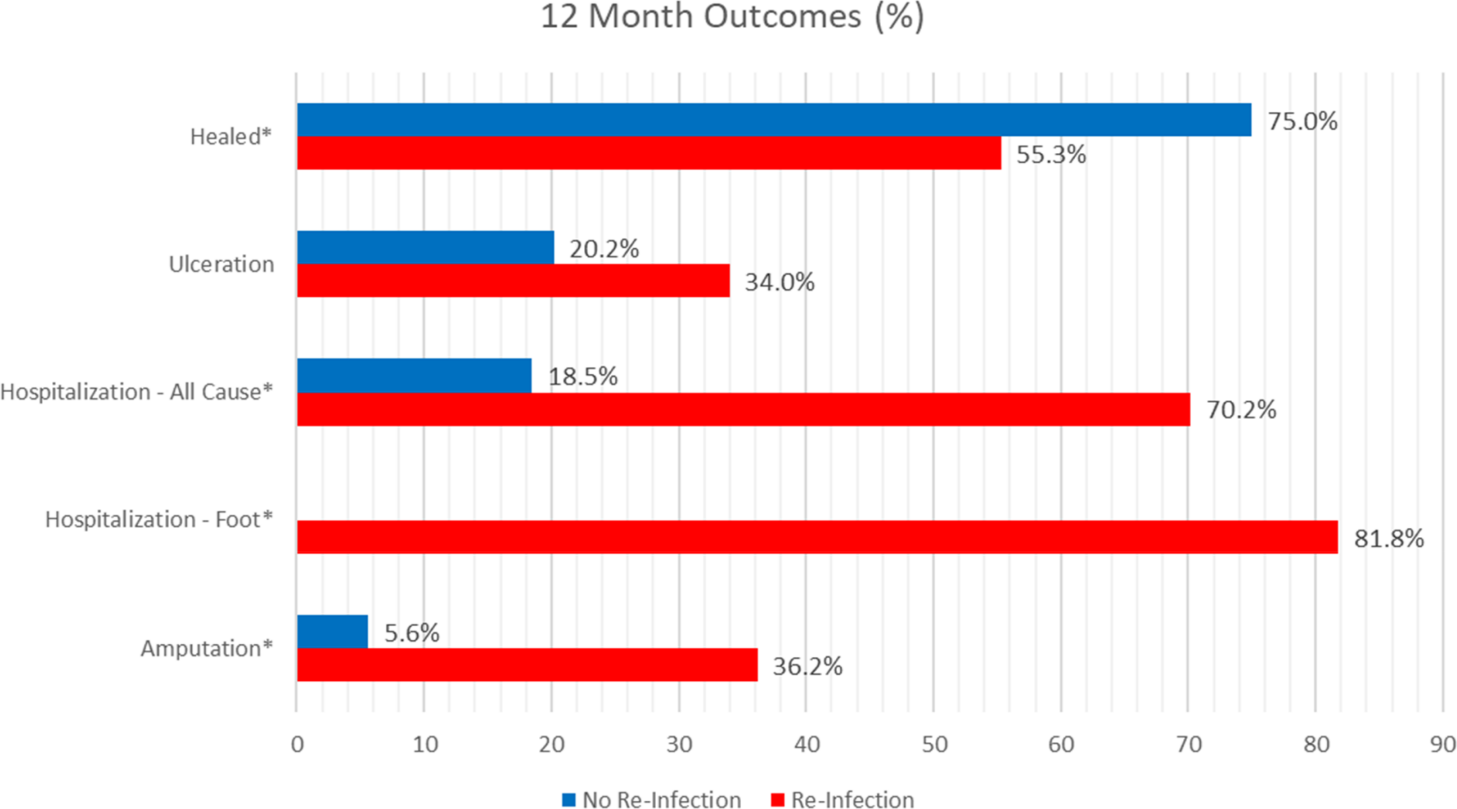
Twelve‐month outcomes in patients with and without re‐infection after diabetic foot osteomyelitis infection. This bar chart compares the 12‐month clinical outcomes in patients with and without re‐infection after osteomyelitis infection. Patients with re‐infection had more complications. Those with re‐infection were 198.8 times more likely to require a foot related hospitalisation (81.8% vs. 0.0%, *p* = 0.001), 10.4 times more likely to have an all‐cause hospitalisation (70.2% vs. 18.5%, *p* = 0.001) 9.4 times more likely to need an amputation (36.2% vs. 5.6%, *p* = 0.001) and less likely to heal (55.3% vs. 75.0%, *p* = 0.01).

Risk factors for re‐infection were Toe Brachial Index <0.40 (25.7% vs. 9.8%, *p* = 0.02), skin perfusion pressure < 40 mmHg (plantar lateral 6.3% vs. 5.9%, *p* = 0.04), wounds healing (55.3% vs. 75.0%, *p* = 0.01), time to heal in days (156.0, 69.5–365 vs. 91.5, 38.8–365, *p* = 0.001) and history of MI (14.9% vs. 3.2%, *p* = 0.005). During the index hospitalisation there was a significant difference in the incidence of amputations (85.1% vs. 70.2%, *p* = 0.04) however, there were no differences in median length of hospitalisation (11.0, 9.0–16.0 vs. 13.0, 10.0–18.0, *p* = 0.24) or in median length of antibiotic duration (23.0, 13.5–47.5 vs. 32.5, 19.0–47.0, *p* = 0.12) (Figure 1).

**FIGURE 2:**
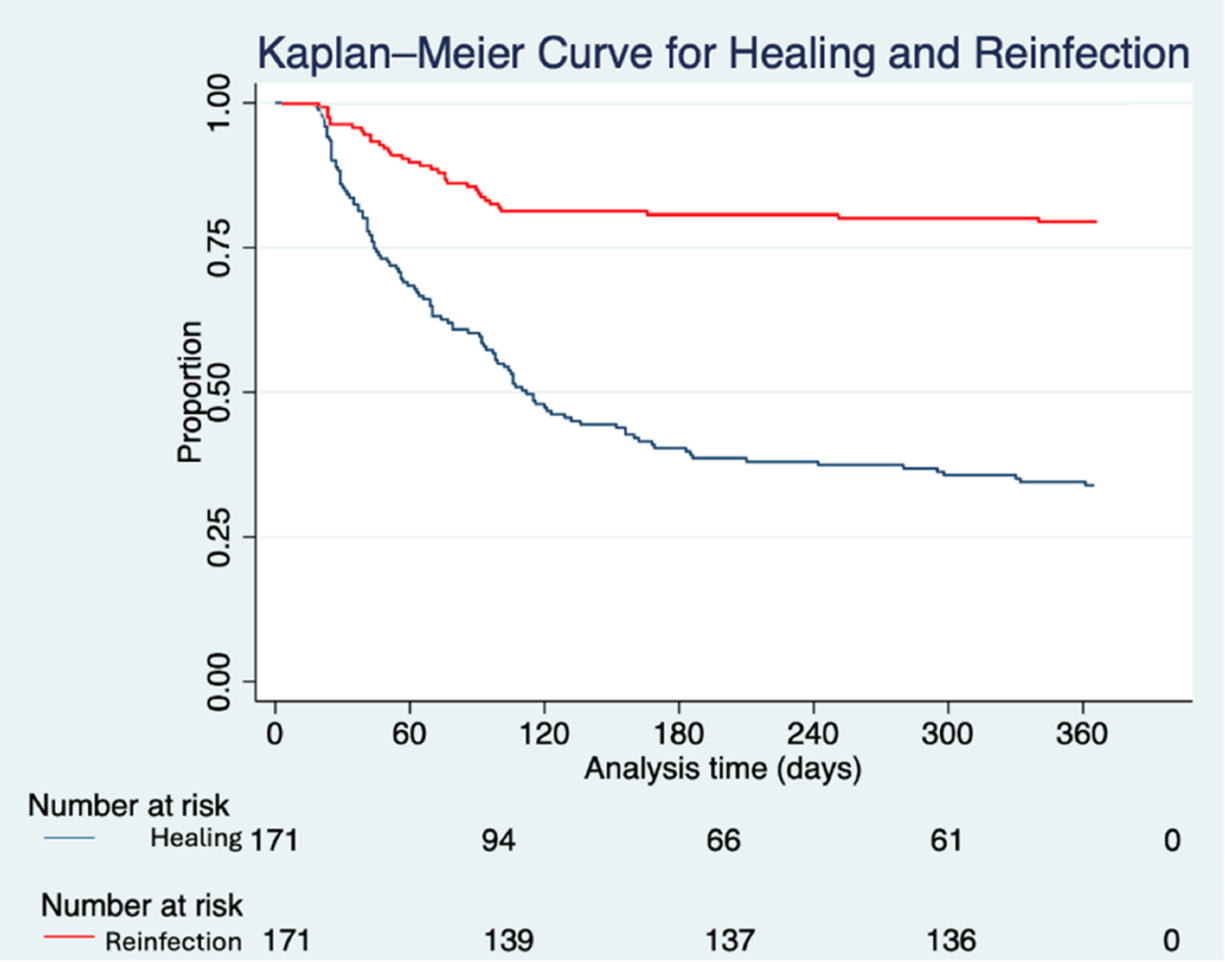
Kaplan Meier survival analysis for healing and re‐infection after diabetic foot osteomyelitis infection. The above presents the Kaplan Meier survival for the primary outcomes healing and reinfection. Healing is represented by the blue line and reinfection by the red line. The mean time to wound healing was 76.4 ± 73.1 days and the mean time to reinfection was 75.7 ± 62.8 days.

During the 12‐month follow‐up, patients with re‐infection experienced more complications compared to patients with no re‐infection (Table 2). Patients with re‐infection were 198.8 times more likely to require a foot related hospitalisation (81.8% vs. 0.0%, *p* = 0.001), 10.4 times more likely have an all‐cause hospitalisation (70.2% vs. 18.5%, *p* = 0.001) and 9.4 times more likely to need an amputation (36.2% vs. 5.6%, *p* = 0.001). Additionally, patients with re‐infection had significantly longer median length of hospitalisation (20.0, 13.5–34.5 vs. 14.0, 10.0–22.0, *p* = 0.003). Patients with re‐infection also experienced significantly longer median length of antibiotic duration (55.0, 35.0–87.0 vs. 46.0, 22.8–68.0, *p* = 0.03). There were no differences in ulceration (34.0% vs. 20.2%, *p* = 0.06). In the Kaplan Meier Survival analysis, the mean time to wound healing was 76.4 ± 73.1 days and the mean time to reinfection was 75.7 ± 62.8 days (Figure 2).

**TABLE 2:** Clinical outcomes in patients with and without re‐infection after diabetic foot osteomyelitis infection.

	Re‐infection (*n* = 47)	No re‐infection (*n* = 124)	(CI 95%)	*p*‐value
Index hospital admission				
Amputation	40 (85.1)	87 (70.2)	2.4 (0.9–5.9)	0.046
Length of stay (days) Median (IQR)	11.0 (9.0–16.0)	13.0 (10.0–18.0)		0.24
Antibiotic days Median (IQR)	23.0 (13.5–47.5)	32.5 (19.0–47.0)		0.12
12‐Month follow‐up				
Ulceration	16 (34.0)	25 (20.2)	2.0 (0.9–4.3)	0.06
Admission—all cause	33 (70.2)	23 (18.5)	10.4 (4.7–22.4)	<0.001
Admission—foot	27 (81.8)	0 (0.0)	198.8 (10.6–3719.1)	<0.001
Amputation	17 (36.2)	7 (5.6)	9.4 (3.6–24.9)	<0.001
Foot amputation	11 (23.4)	1 (0.8)	11.0 (1.1–114.0)	0.03
Leg amputation	6 (12.7)	6 (4.8)	0.1 (0.0–0.9)	0.03
Total length of stay (days) Median (IQR)	20.0 (13.5–34.5)	14.0 (10.0–22.0)		0.003
Total antibiotic days Median (IQR)	55.0 (35.0–87.0)	46.0 (22.8–68.0)		0.03

*Note*: Descriptive variables are presented as *N* (%).

*Note*: Continuous variables as mean (standard deviation) or median (IQR) for non‐normally distributed data.

## 4 DISCUSSION

The results of this study show the incidence and risk factors for re‐infection in a prospective cohort of diabetic patients treated for diabetic foot osteomyelitis. We have not been able to identify other studies that report risk factors for re‐infection. Re‐infection is an important consideration, because it essentially restarts the clock and increases the likelihood of complications. During the follow‐up period, 27.5% of study subjects developed infection. Markers for peripheral vascular disease (TBI < 0.4 and skin perfusion pressure), the proportion of wounds that healed, prolonged time to heal, and history of MI were associated with re‐infection. Many of the traditional measures of perfusion were not associated with re‐infection such as ABIs. We were surprised that other variable were not related to re‐infection such as social determinants of health, sepsis at the time of admission, co‐morbidities, medications, and the presence of residual osteomyelitis after surgery.

Our incidence of re‐infection was lower than we expected. There are several randomised clinical trials for various products to treat relatively small diabetic foot ulcers (3 cm^3^) that report infection rates of 14%–32% in subjects in the standard of a care arm of 12‐week studies.^14^, ^15^, ^16^, ^17^ Because the infection rate was high in such a short period of time, we expected higher rates of re‐infection in people with complex wounds over a year. In addition, our subjects had a high rate of residual osteomyelitis after their first surgery (52.0%), which has been associated with longer duration of antibiotic treatment, re‐infection, and amputation in other studies.^18^, ^19^, ^20^, ^21^ However, residual bone infection after surgery was not an important risk factor in this study.

Other reports of re‐infection after hospitalisation and surgery for complex foot infections range from 27.0% to 47.3%.^7^, ^22^ In patients with osteomyelitis that can be treated without surgery, the re‐infection incidence ranges from 27.5% to 36.0% per year.^23^, ^24^ Even though re‐infection is common, we have been unable to identify published studies that evaluate risk factors for re‐infection.

There are important limitations to this study. Certainly, there is selection bias in this study which can produce incorrect estimates of the treatment effect. All study subjects were admitted to the hospital for moderate and severe diabetic foot infections requiring surgery. Patients had a combination of soft tissue and bone infection that required surgery. This is another limitation of the study as all the subjects had infections that required surgery. Thus, our results are not generalizable to DFUs that are classified as mild infections or moderate/severe infections that do not undergo surgery. Other studies that evaluate people with chronic osteomyelitis without soft tissue infection present with a different scenario and probably have different clinical outcomes. When there is not an underlying abscess, there is time for advanced imaging, biopsy and pathogen directed antibiotic therapy.^13^, ^24^

## 5 CONCLUSION

Re‐infection in diabetic patients after discharge from hospital for osteomyelitis is common (27.5%). The risk factors for re‐infection included toe brachial indices <0.4, and skin perfusion pressure measurement <40 mmHg, wound healing, and prolonged time to heal and history of myocardial infarction. People with re‐infection are more likely to have more hospitalizations, more amputations, longer treatment with antibiotics, and longer hospitalizations.

## AUTHOR CONTRIBUTIONS

LAL formulated study design and manuscript preparation. MCR performed data collection. MCR, LAL and ANT interpreted and analysed the data. MJS, MCS, TLC, BN critically reviewed manuscript. All authors discussed the results and contributed to the final manuscript.

## FUNDING INFORMATION

This work was supported by the American Diabetes Association (Translational Research Award 7‐14‐TS‐20) and Cardinal Health.

## CONFLICT OF INTEREST STATEMENT

The authors declare no conflicts of interest.

## Data Availability

The data that support the findings of this study are available from the corresponding author upon reasonable request.
